# Characterization of Odorous Compounds (VOC and Carbonyl Compounds) in the Ambient Air of Yeosu and Gwangyang, Large Industrial Areas of South Korea

**DOI:** 10.1155/2014/824301

**Published:** 2014-09-17

**Authors:** Young-Kyo Seo, Lakshmi Narayana Suvarapu, Sung-Ok Baek

**Affiliations:** Department of Environmental Engineering, Yeungnam University, Gyeongsan-si 712 749, Republic of Korea

## Abstract

Odorous compounds play an important role in air pollution in industrial areas and the residential areas surrounding them. This study measured the odorous volatile organic compounds (VOC) and carbonyl compounds at Yeosu and Gwangyang, two large industrial areas of South Korea, during four seasons of 2008-2009. Along with these two cities, the same odorous compounds were measured at Suncheon, which was selected as a control site. The concentrations of VOC and carbonyl compounds that were listed as odorous air pollutants by the Ministry of Environment of South Korea are discussed. Benzene and formaldehyde were included in the target analytes because of their carcinogenic nature. Most researchers only examined the concentration of odorous compounds in ambient air but the present study evaluated the odor intensity, which is a new parameter that will help better understand the precise odor perceived by people. This paper describes the seasonal variations and spatial distribution of the above-mentioned odorous compounds at the specified sites. Pearson correlation coefficients between the odorous compounds and other air pollutants, such as ozone, CO, SO_2_, NO_2_, and PM_10_, and meteorological conditions, such as temperature and wind speed, provide the source information of odorous VOC and carbonyl compounds.

## 1. Introduction

The monitoring of odorous compounds in ambient air is an important task to environmental researchers because of the presence of some toxic volatile organic compounds (VOC) and carbonyl compounds in odorous compounds. The VOC and carbonyl compounds present in malodors have adverse effects on the air quality in the surrounding areas of the sources [1] as well as on health of the people residing near the sources. The major sources for odorous compounds include agricultural waste, food processing industries [2, 3], composting of municipal solid wastes [4], livestock production industries [5], semiconductor industries [6], and the incomplete combustion of hydrocarbon fuels in a range of industries [7]. The use of ethanol in industries also results in the release of odorous carbonyl compounds into the atmosphere [8]. Therefore, many countries are struggling to develop effective odor regulations or guidelines to decrease their concentrations in ambient air [2]. In recent years, many researchers have reported the concentrations of odorous compounds in the atmosphere in Korea and around the world with respect to their hazardous and/or toxic nature to humans [7–12]. In South Korea, the Ministry of Environment identifies 22 compounds as malodourous in the atmosphere [13]. Among these 22 malodorous compounds, 50% belong to VOC (toluene, styrene,* m,p,o*-xylenes, methyl isobutyl ketone, and ethyl acetate) and carbonyl compounds (acetaldehyde, propanal, n-butanal, n-valeraldehyde,* i*-valeraldehyde, and methyl ethyl ketone).

In addition to odor, the carbonyl compounds have also attracted the attention of researchers to measure their concentrations in ambient air because of their ozone formation potential by means of photochemical reactions and their adverse health effects on humans [14, 15].

**Figure 1:**
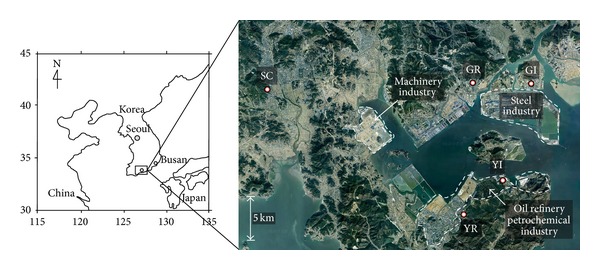
Location of the sampling sites at Yeosu, Gwangyang, and Suncheon.

In the present study, ambient air samples were collected to measure the concentrations of odorous compounds including VOC and carbonyl compounds in two large industrial cities of South Korea, namely, Yeosu and Gwangyang. To compare the concentrations of the selected compounds at these cities, Suncheon, which is a purely residential site and almost 25 km away from industrial complexes, was chosen as the control site (Figure 1).

Yeosu (34°44′N 127°44′E), which is situated in Jeolla province, South Korea, is an important industrial city. The population is approximately 300,000 according to the 2009 census and has an area of 503.33 km^2^. This city has more tourist attractions as an ocean resort and is also close to the Yeocheon Industrial Complex. This importance made this city a host to the 2012 World Expo (http://en.wikipedia.org/wiki/Yeosu). The major industries located in this city are related to oil refineries and petrochemicals. In Yeosu, ambient air samples were collected at two sites: pure industrial site and a residential site near the industrial complexes. These two sites were 5 km from each other (Figure 1). Gwangyang (34°56′N 127°41′E) is also important and large industrial cities of South Korea located in South Jeolla province of the country. This is the home of the POSCO (Pohang Steel Company), which is one of the world's largest steel companies. This city has a population and area of approximately 150,000 and 309.4 Km, respectively (http://en.wikipedia.org/wiki/Gwangyang, accessed on July 10, 2014). The major industries located in this city are related to steel companies. In the present study, ambient air samples were collected at two sites of Gwangyang representing industrial and residential areas separated by a distance of 5 km (Figure 1).

This paper reports the measured odorous VOC and carbonyl compounds at Yeosu and Gwangyang, which are two important industrial cities of South Korea during the four seasons of 2008-2009. The concentrations of benzene and formaldehyde were also measured, even though they are not listed as odorous compounds in South Korea because of their carcinogenic nature [16, 17]. The concentrations of odorous VOC and carbonyl compounds measured at these industrial cities were also compared with those measured at Suncheon, a nonindustrial control site. The odor intensity of each VOC and carbonyl compounds was also evaluated to determine the precise odor impact of these compounds on humans. This is the first comprehensive study in Korea regarding the monitoring of VOC and carbonyl compounds in ambient air in terms of the huge number of samples and variety of odorous compounds.

## 2. Materials and Methods

### 2.1. Sampling Sites and Sampling Periods

Ambient air samples were collected for an analysis of VOC and carbonyl compounds at Yeosu Industrial (YI), Yeosu Residential (YR), Gwangyang Industrial (GI), Gwangyang Residential (GR), and Suncheon Control (SC) sties, for 10 consecutive days during spring (May 20–29), summer (August 5–14), and fall (October 17–26) of 2008 and winter (January 13–22) of 2009. At all sampling sites and for all seasons, two samples were collected manually for an analysis of carbonyl compounds for a 2 h duration and six samples were collected using an automatic continuous sampler for VOC analysis at a 4 h duration on each sampling day. On the other hand, at the YR site, only two VOC samples were collected manually per day in summer and spring and two samples per day in summer at the GR site. This is largely due to the instrumental availability during the specified period. According to the Korean Meteorological Administration (http://web.kma.go.kr/eng/index.jsp, accessed on July 10, 2014), the average temperatures observed in spring, summer, fall, and winter at Yeosu were 20.9°C, 28.2°C, 19.4°C, and 3.1°C, respectively. The mean temperatures observed in spring, summer, fall, and winter at Gwangyang were 20.5°C, 27.5°C, 17.2°C, and 2.9°C, respectively.

### 2.2. Sampling and Analysis of VOC and Carbonyl Compounds

Stainless steel tubes (1/4′′ × 9 cm, Perkin Elmer, UK), packed with 120 mg Carbograph 2TD and 280 mg of Carbograph 1TD (40/60 mesh, Markes, UK), a low flow rate pump equipped with a mass flow controller (Flec, Chematec Inc., Denmark), and a sequential automatic tube sampler (STS 25, Perkin Elmer, UK) were used to determine the concentration of odorous VOC at the specified sites. The sampling of VOC was carried out at a flow rate of 100 mL/min for 4 h per each tube. Preconditioning of the adsorbent tubes was performed before sampling at the temperature of 250°C for 2 h with helium as the carrier gas. The performance of the adsorbent tubes used in this study was tested previously by the author's research group [37, 38].  Table 1 lists the analytical and operating conditions for this system. The total analytical procedure followed in the analysis of VOC was described in detail in a previous research paper [39].  Table 2 lists the physical-chemical characteristics of both odorous VOC and carbonyl compounds.

For the qualitative and quantitative determination and analysis of carbonyl compounds, the calibration of carbonyl-DNPH mixture (Supelco Inc., USA) was performed with the standard working solutions diluted as 0.25, 0.5, 1.0, 3.0, and 15.0 *μ*g/mL in the case of formaldehyde. The commercial standard mixture did not contain the methyl ethyl ketone, which was calibrated separately using an individual standard after dilution.

The sampling of carbonyl compounds was performed using DNPH cartridges (C18 Sep-Pak short-body cartridges coated with DNPH, Supelco Inc., USA). The interference of ozone during sampling was removed by connecting a polypropylene tube filled with KI (~1.4 g) at the front side of the DNPH cartridge. During sampling, a total volume of 120 L of ambient air was collected at a flow rate of 1 L/min using a sampling pump. The resulting samples were kept in a refrigerator prior to the analysis of carbonyl compounds through the solvent extraction process. The DNPH derivatives were then extracted with 4 mL of acetonitrile and the extracted solution was transferred into an amber vial (to avoid contact with sunlight), sealed tightly with Teflon tape and stored in refrigerator. All glassware used during the solvent extraction process was prewashed with acetonitrile and dried over 60°C prior to use. The extracted DNPH derivatives were analyzed by HPLC with a UV detector at 360 nm [40].  Table 3 lists all the operating conditions used in HPLC analysis of the carbonyl compounds.

### 2.3. Quality Control and Quality Assurance

Detailed calibration process of VOC followed in this study was prescribed in a previous research paper [39]. The precision of the present method for an analysis of VOC was evaluated by the relative standard deviation (RSD) of the retention time and peak area of a known amount of VOC standard injected into each adsorbent tube. Overall, the RSDs for the peak area appeared to be <8%, whereas those for the retention time were <0.5%. The detection limit for the present method was evaluated according to the US EPA TO-17 method protocol [41] and was obtained as 0.01–0.06 ppb, depending on the individual VOC.

**Figure 2:**
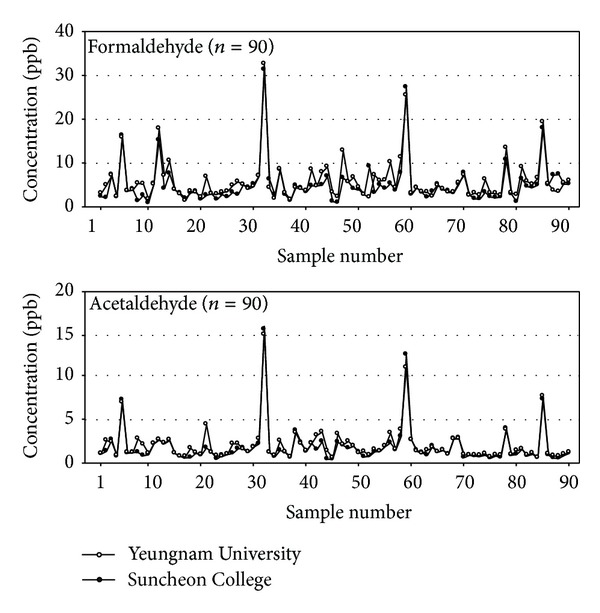
Concentrations of formaldehyde and acetaldehyde obtained during the analysis at Yeungnam University and Suncheon College (interlab comparison).

The repeatability of the present method was evaluated using the relative standard deviation (RSD) of the peak areas for a standard mixture (0.25 *μ*g/mL). The RSD of the present method within a day appeared to be less than 3.5% and between days it was less than 8.0%. The detection limit for the analysis of carbonyl compounds in the present method was evaluated using the guidelines of the US EPA [42] and was obtained as 1.1~2.3 ng/mL, which is equal to 0.01~0.02 ppb (as the concentration in the air based on the total volume of 120 L). The accuracy of the present analytical method was tested by an interlaboratory comparison of the analytical data of the concentrations of carbonyl compounds. For an interlaboratory comparison, 90 carbonyl samples were taken in random and analyzed in the author's laboratory (Yeungnam University, Korea) and another laboratory (Suncheon College, Korea).  Figure 2 shows the concentrations of both formaldehyde and acetaldehyde during interlaboratory analysis which is a similar trend for all samples in two laboratories.  Table 4 lists the statistical parameters of the interlaboratory comparison, which indicates an excellent correlation between the data obtained in the two laboratories.

## 3. Results and Discussion

### 3.1. Occurrence and Distribution of VOC and Carbonyl Compounds

Table 5 lists the detection frequencies and the mean, median, minimum, and maximum concentrations of the odorous VOC measured at Yeosu Industrial (YI), Yeosu Residential (YR), Gwangyang Industrial (GI), Gwangyang Residential (GR), and Suncheon Control (SC) sites. Benzene, toluene, and* m,p,o*-xylenes were found with detection frequencies in the range from 95 to 100% at all sites. With the exception of the YI site, styrene and methyl isobutyl ketone were found at detection frequencies below 30% and 9%, respectively, at all other sites. Overall, at Yeosu industrial site, all the VOC were observed at higher detection frequencies than the other sites. From Table 5, it is clear that the most abundant odorous VOC found at all these sites was toluene followed by* m,p,o*-xylenes. In addition to odorous VOC, benzene, the most important and carcinogenic VOC, was found at higher levels at all these sites than in any other VOC measured. The national air quality standard for benzene, as an annual average concentration, was established as 1.5 ppb, in Korea. The concentration of benzene was found to be higher than the Korean standard level at the industrial sites (YI and GI) and lower at the residential areas (YR and GR) and control site (SC). Regarding the odorous VOC, the least abundant compounds were methyl isobutyl ketone and styrene.

Table 6 lists the detection frequencies and the mean, median, minimum, and maximum level concentrations of odorous carbonyl compounds measured at YI, YR, GI, GR, and SC sites. Formaldehyde and acetaldehyde were found at higher detection frequencies (96–100%) at all sites followed by methyl ethyl ketone (73–91%). The compounds, such as butyraldehyde,* i*-valeraldehyde, and* n*-valeraldehyde, were detected with the lowest frequencies at all sites. Regarding the concentrations of odorous carbonyls, acetaldehyde, methyl ethyl ketone, and propionaldehyde were found to be higher in all sampling sites than the others. On the other hand, the concentration of formaldehyde found was higher than any other carbonyl compound at all sites. Butyraldehyde,* i*-valeraldehyde, and* n*-valeraldehyde were observed at lower concentrations at all sites.

### 3.2. Spatial Distribution of VOC and Carbonyl Compounds

**Figure 3:**
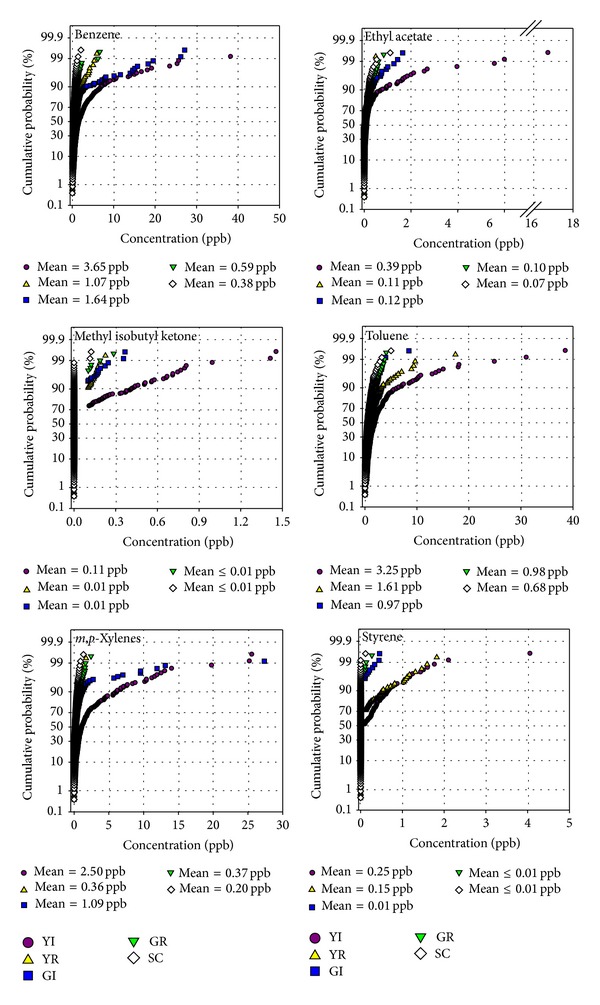
Cumulative probability of the distributions of the concentrations of VOC.

**Figure 4:**
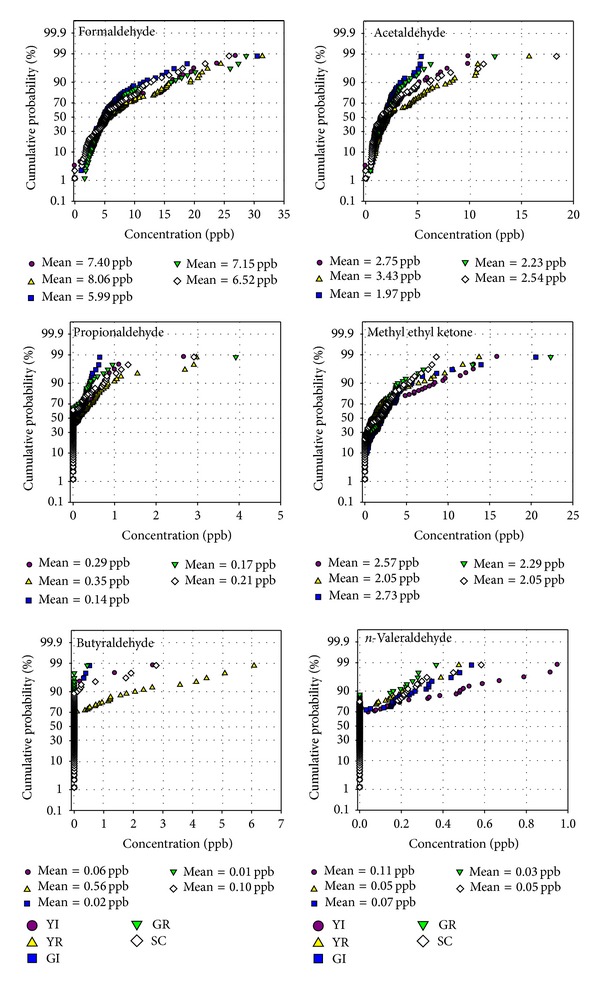
Cumulative probability of distributions of the concentrations of carbonyl compounds.

Figures 3 and 4 present the cumulative probabilities of distributions of the concentrations of the VOC and carbonyl compounds measured at all five sites (YI, YR, GI, GR, and SC). All the measured VOC showed higher levels at the Yeosu Industrial (YI) site and lower levels at the control site (SC) compared to the other sites. Benzene and* m,p-*xylenes were observed to be higher at the industrial sites (YI and GI) than the residential sites. Toluene and styrene showed higher levels at the Yeosu industrial and residential sites than the other sites. The concentration of benzene at YI site was almost 2–9 times higher than the other sites. Regarding carbonyl compounds, a few of them were higher at the residential site (YR) than at the other sites. Carbonyl compounds are not only emitted from primary sources but also formed by photochemical reactions in the atmosphere (secondary sources). This might explain the higher concentrations of few carbonyls at residential sites than the industrial sites. The residential areas were affected from the emissions of industrial and traffic sources.

### 3.3. Seasonal Variations in Concentrations of VOC and Carbonyl Compounds

**Figure 5:**
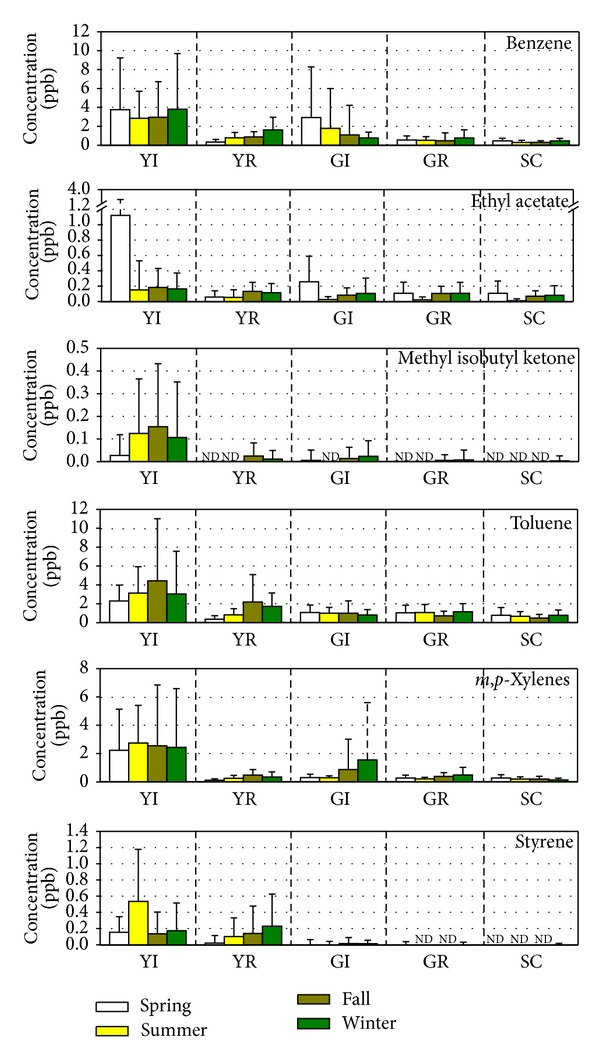
Seasonal variations of concentrations of VOC.

**Figure 6:**
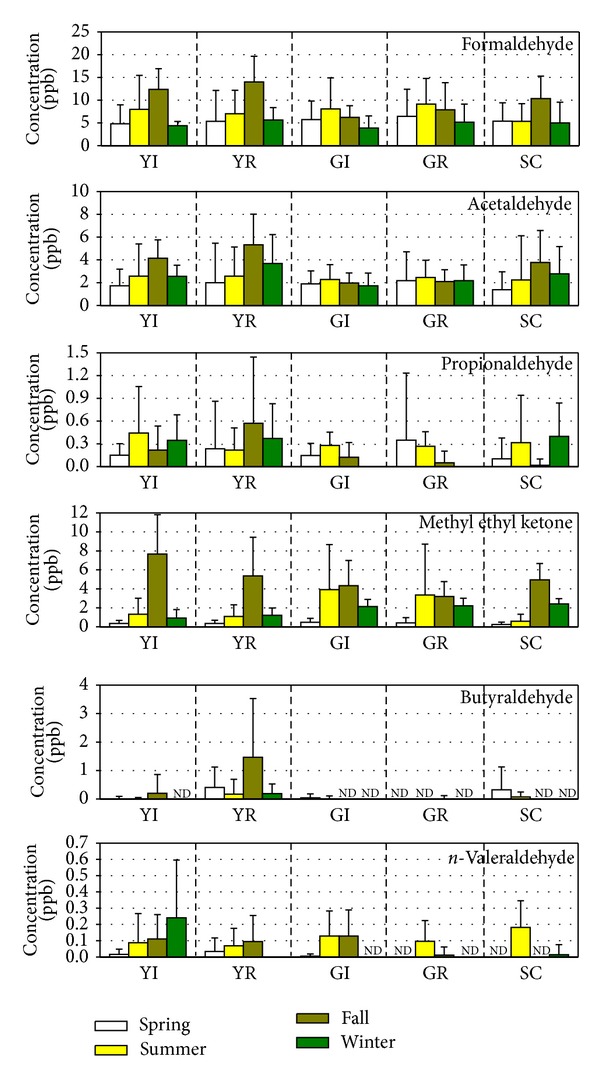
Seasonal variations of the concentrations of carbonyl compounds.

To understand the seasonal influence on the concentrations of VOC and carbonyl compounds, their concentrations were measured during four seasons of the year and are shown in Figures 5 and 6, respectively. From this study, the concentrations of VOC and carbonyl compounds were not much influenced by the seasons. Owing to multisources and secondary reactions in the atmosphere, the concentrations of these compounds did not show constant variations in different seasons.

### 3.4. Odor Intensity of VOC and Carbonyl Compounds

**Figure 7:**
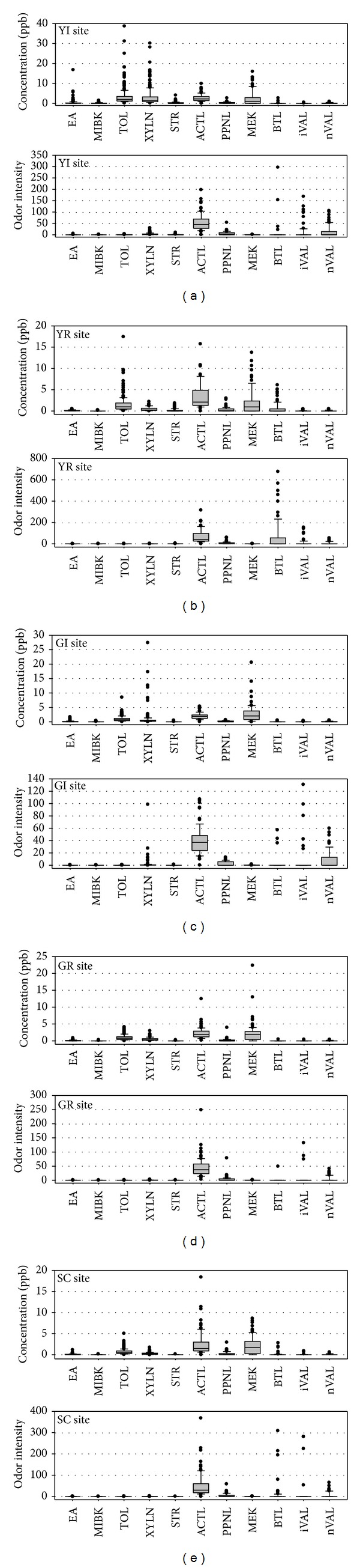
(a) Concentration and odor intensity of the odor compounds at YI site. (b) Concentration and odor intensity of the odor compounds at YR site. (c) Concentration and odor intensity of the odor compounds at GI site. (d) Concentration and odor intensity of the odor compounds at GR site. (e) Concentration and odor intensity of the odor compounds at SC site.

Most researchers measured the concentrations of the VOC and carbonyl compounds in ambient air. On the other hand, the concentrations may not indicate their precise impact on the people's health, particularly odorous compounds. A new parameter, odor intensity, was introduced to evaluate the accurate odor impact on people when they perceived these compounds. The odor intensity of a compound can be calculated by dividing the target analyte concentration with the standard (reference) odor intensity value of that compound. The reference values were taken from the Japanese Odor Control Law (http://www.env.go.jp/en/laws/air/odor/opm.html, accessed on July 10, 2014) to calculate the odor intensities of the VOC and carbonyls. Figures 7(a), 7(b), 7(c), 7(d), and 7(e) show the concentrations and odor intensities of odorous compounds measured at the YI, YR, GI, GR, and SC sites, respectively. In an odor intensity point of view, the maximum intensity is more significant than the mean or median value. From these figures, toluene, xylenes, acetaldehyde, and methyl ethyl ketone were found in higher concentrations, and butyraldehyde,* i*-valeraldehyde, and* n*-valeraldehyde were found at lower concentrations at most sites. Regarding the odor intensity; however, the compounds found at lower concentrations (butyraldehyde,* i*-valeraldehyde, and* n*-valeraldehyde) had more odor impact than the compounds with higher concentrations. In the case of acetaldehyde, both the concentrations and odor intensity were high.

### 3.5. Correlations of the VOC, Carbonyl Compounds, and Air Quality/Meteorological Data

Pearson correlation analysis was performed to examine the factors affecting the concentrations of VOC and carbonyl compounds at the study areas. Tables 7(a) and 7(b) list the correlation coefficients among the VOC and VOC to the other criteria air pollutants and meteorological conditions. In the case of VOC, aromatic VOC, such as, toluene,* m,p*-xylenes, and* o*-xylene, were correlated with each other and indicated a common source. The meteorological conditions, such as temperature and wind speed, showed a low correlation with the VOC indicating that they do not affect the concentrations of VOC at specified sites. Benzene showed a good correlation with CO at the residential sites (0.592 and 0.448 at YR and GR, resp.), indicating that combustion is a major of benzene. Similarly NO_2_ and SO_2_ showed a good correlation with aromatic VOC, suggesting that the fossil fuel combustion and other combustion processes in industries were the major sources of these compounds.

Tables 8(a) and 8(b) present the correlation coefficients among carbonyl compounds and carbonyls with other criteria air pollutants and meteorological conditions. The correlations between the carbonyl compounds and to the combustion related compounds, such NO_2_, SO_2_, and CO, did not show any significant relationships among them, indicating that most of the carbonyl compounds came from fugitive emissions and not from combustion. In the formaldehyde case, it was well correlated with ozone during summer at four sites (0.648, 0.401, 0.481, and 0.709 at YI, YR, GI, and GR, resp.), suggesting that formaldehyde was produced by secondary photochemical reactions, such as ozone [43]. The meteorological conditions were not correlated or negatively correlated with the carbonyls, indicating that the concentrations of carbonyls were unaffected by the seasonal influence. The concentrations of VOC were affected by the wind direction while the same pattern was not observed in case of carbonyls. The concentrations of VOC were affected by the wind direction while the same pattern was not observed in case of carbonyls. The sources of odorous carbonyls seem to be much complex than VOC, and we estimated that they were mostly emitted by local sources and oxidation of primary pollutants (as an example, acetaldehyde from alcohols). In this respect, it is an important task to find out the local sources of carbonyls. At present we do not have much information about the local sources of carbonyls at specified study sites.

### 3.6. Comparison of the Concentrations of VOC and Carbonyl Compounds of the Present Study with the Others Reported Worldwide

The concentrations of VOC and carbonyl compounds measured in this study were compared with those of other cities in the world and are presented in Tables 9 and 10, respectively. For this purpose, present data was compared with other data measured mainly at industrial and residential sites. The concentration of benzene found in Yeosu Petrochemical Industrial Site is lower than that reported for other petrochemical industrial sites at Mumbai (India) and Kaohsiung (Taiwan) and higher than those reported for Dunkerque (France), Rem-Wu (Taiwan), and Yokohoma (Japan). On the other hand, the benzene concentration found at Gwangyang Steel Industrial Site was lower than that of most steel industrial sites compared except for the Pohang (South Korea) Steel Industrial Site. The toluene to benzene ratio (T : B) found in the present study was in the range of 0.59–1.79.

The concentration of formaldehyde (group 1 carcinogen) reported in the present study was lower than that reported at Kolkata (India), Guangzhou (China), and Ansan (South Korea), and higher than at Gumi (South Korea), Rome (Italy), and Mexico City (Mexico). Most researchers worldwide reported very low concentrations of* i*-valeraldehyde and* i*-valeraldehyde and butyraldehyde (except Ansan, South Korea). On the other hand, although they exist at low concentrations, their odor intensity was higher than other higher abundant carbonyl compounds.

### 3.7. Health Implications of Benzene and Formaldehyde Levels from This Study

The WHO has no standard for benzene, since benzene is known to be nonthreshold pollutant. Instead, the WHO [44] provides a unit risk for benzene, which is 6 × 10^−6^. This means that the life time exposure to 1 *μ*g m^−3^ (c.a. 0.31 ppb) of benzene will cause six cases of leukemia per one million of the population. According to the present results, the mean concentration of benzene was 1.07 ppb (YR) and 0.59 (GR), which may cause six and two leukemia cases among the life-time residents in Yeosu residential area and Gwangyang residential areas, respectively. The unit risk for formaldehyde is 1.3 × 10^−5^. This suggests that the life time exposure to 1 *μ*g m^−3^ (c.a. 0.79 ppb) of formaldehyde will cause one case of cancer per one million of the population. The mean concentrations of formaldehyde found in the present study were 8.06 ppb (YR), and 7.15 ppb (GR), which may cause forty and seventeen cancer cases among the life-time residents in Yeosu residential area and Gwangyang residential areas, respectively. This prediction is based solely on the concentrations of the respective compounds measured in this study and the residents of these cities if exposed for a lifetime.

## 4. Conclusion

This paper reported the concentrations of odorous VOC and carbonyl compounds at large industrial areas of South Korea. In this study, the levels of few carbonyls were higher in residential areas near industrial areas. This indicates the influence of industrial sources on the air quality of residential areas in close proximity. Few of the carbonyls were found to be higher in residential areas than in industrial areas; more focus on the determination of the source profiles of these particular compounds is needed. The residential areas provide some sources for these compounds and/or the secondary reactions may also be responsible. In the present study, the odor intensity of the VOC and carbonyls, which gives an accurate impact on the people when they perceived these compounds, was determined. In addition to the odorous compounds, carcinogens, such as, benzene and formaldehyde, were found to be higher at industrial sites than the Korean National Standard Level Concentrations.

## Figures and Tables

**Table 1:** Analytical conditions for VOC with thermodesorption GC/MSD.

Thermal desorber UNITY/ULTRA (Markes, UK)		GC/MSD (HP6890/5973, Hewlett Packard, USA)	
Oven temp.	300°C	GC column	Rtx-1 (0.32 mm, 105 m, 1.5 *μ*m)
Desorb time	10 min	Initial temp.	50°C (10 min)
Desorb flow	50 mL/min	Oven ramp rate	5°C/min
Cold trap holding time	5 min	Final temp.	250°C (5 min)
Cold trap high temp.	320°C	Post run	250°C (5 min)
Cold trap low temp.	−10°C	Column flow	1.13 mL/min
Cold trap packing	Tenax TA/carbopack B	Detector type	Quadropole
Min. pressure	12 psi	Q-pole temp.	150°C
Inlet split	No	MS Source temp.	230°C
Outlet split	10 mL/min	Mass range	35~300 amu
Valve and line temp.	180°C	Electron energy	70 eV

**Table 2:** Physicochemical characteristics of odor compounds in this study.

Compounds	Abb.	CAS No.	Odorous compounds		MW (g/mol)	Bp (°C)
			Korea	Japan		
Benzene	BZ	71-43-2	—	—	78.11	80.1
Ethyl acetate	EA	141-78-6	—	○	88.11	77.1
Methyl isobutyl ketone	MIBK	108-10-1	○	○	100.16	117.0
Toluene	TOL	108-88-3	○	○	92.14	111.0
*m*, *p*-Xylenes	mpXYLN	108-38-3 106-42-3	○	○	106.16	139.0
*o*-Xylene	oXYLN	95-47-6	○	○	106.17	144.4
Styrene	STR	100-42-5	○	○	104.15	145.0
Formaldehyde	FRML	50-00-0	—	—	30.03	−19.0
Acetaldehyde	ACTL	75-07-0	○	○	44.05	20.2
Propionaldehyde	PPNL	123-38-6	○	○	58.08	46.0
Methyl ethyl ketone	MEK	78-93-3	○	—	72.11	79.6
Butyraldehyde	BTL	123-72-8	○	○	72.11	74.8
*i*-Valeraldehyde	iVAL	590-86-3	○	○	86.13	92.0
*n*-Valeraldehyde	nVAL	100-62-3	○	○	86.13	102.0

**Table 3:** Operating conditions for the HPLC analysis of carbonyl compounds.

Operating parameter	Specifications and conditions
HPLC system	Shimadzu SCL-6B with Shimadzu SPD-6 AV UV/VIS detector at 360 nm
Analytical column	Shim-Pack CL-ODS (M) 4.6 mm × 150 mm with a C_18_ guard column
Mobile phase	A: acetonitrile 100 (V), B: water/acetonitrile/tetrahydrofuran 50/45/5 (v/v)
Gradient elution	100% for B for 5 min, and then 60 : 40% A : B for 15 min, finally 100% A for 50 min
Flow rate and injection volume	1.0 mL/min and 20 *μ*L injection

**Table 4:** Interlaboratory comparison of the concentration of same carbonyl compounds analyzed at author's laboratory (Yeungnam University, YNU) and another laboratory (Suncheon College, SCC).

Compounds	Sample number	MDP^(1)^ (%)	MRE I^(2)^ (%)	MRE II^(3)^ (%)	*R* ^ (4)^	*t*-test (*P* value)	Paired *t*-test (*P* value)
Formaldehyde	90	27.8	23.9	37.8	0.94^∗(5)^	0.2	0
Acetaldehyde	90	18.5	15.3	25.6	0.97∗	0.46	0

^(1)^MDP (mean duplicate precision, %) = (1/*n*)∑_*n*=1_^*n*^(|YNU − SCC|/Mean) × 100 (%).

^(2)^MRE I (mean relative error, %) = (1/*n*)∑_*n*=1_^*n*^(|YNU − SCC|/YNU) × 100 (%).

^(3)^MRE II (mean relative error, %) = (1/*n*)∑_*n*=1_^*n*^(|YNU − SCC|/SCC) × 100 (%).

^(4)^ *R*: correlation coefficient.

^(5)∗^Correlation coefficients are significant at a level of 0.05.

**Table 5:** Statistical summaries of the VOC at different sites in the present study.

Sites	VOC	Detection frequency	Mean (ppb)	S.D. (ppb)	Median (ppb)	Min (ppb)	Max (ppb)
YI (*n* = 234)	Ethyl acetate	79%	0.39	1.29	0.11	N.D.	16.75
	Benzene	100%	3.65	6.86	1.75	0.14	38.31
	Methyl isobutyl ketone	26%	0.11	0.23	N.D.	N.D.	1.46
	Toluene	100%	3.25	4.40	2.01	0.06	38.61
	*m*, *p*-Xylenes	100%	2.50	3.57	1.17	0.06	25.61
	Styrene	48%	0.25	0.44	N.D.	N.D.	4.07
	*o*-Xylene	100%	0.58	0.65	0.34	0.02	4.47

YR (*n* = 160)	Ethyl acetate	76%	0.11	0.12	0.07	N.D.	0.49
	Benzene	100%	1.07	1.02	0.75	0.04	5.93
	Methyl isobutyl ketone	9%	0.01	0.04	N.D.	N.D.	0.23
	Toluene	100%	1.61	2.09	0.98	0.05	17.41
	*m*, *p*-Xylenes	99%	0.36	0.35	0.20	N.D.	1.71
	Styrene	29%	0.15	0.34	N.D.	N.D.	1.82
	*o*-Xylene	98%	0.10	0.09	0.07	N.D.	0.47

GI (*n* = 239)	Ethyl acetate	62%	0.12	0.22	0.05	N.D.	1.64
	Benzene	100%	1.64	3.83	0.40	0.06	27.12
	Methyl isobutyl ketone	6%	0.01	0.05	N.D.	N.D.	0.37
	Toluene	100%	0.97	0.87	0.75	0.04	8.45
	*m*, *p*-Xylenes	99%	1.09	5.78	0.28	N.D.	27.36
	Styrene	4%	0.01	0.05	N.D.	N.D.	0.46
	*o*-Xylene	97%	0.28	1.22	0.08	N.D.	16.06

GR (*n* = 199)	Ethyl acetate	73%	0.10	0.13	0.06	N.D.	0.83
	Benzene	100%	0.59	0.72	0.40	0.07	6.66
	Methyl isobutyl ketone	3%	<0.01	0.03	N.D.	N.D.	0.29
	Toluene	100%	0.98	0.77	0.78	0.01	4.10
	*m*, *p*-Xylenes	98%	0.37	0.36	0.23	N.D.	2.43
	Styrene	2%	<0.01	0.02	N.D.	N.D.	0.27
	*o*-Xylene	95%	0.11	0.10	0.08	N.D.	0.52

SC (*n* = 240)	Ethyl acetate	53%	0.07	0.11	0.03	N.D.	1.11
	Benzene	100%	0.38	0.25	0.33	0.04	2.11
	Methyl isobutyl ketone	1%	<0.01	0.01	N.D.	N.D.	0.12
	Toluene	100%	0.68	0.60	0.52	0.01	5.02
	*m*, *p*-Xylenes	97%	0.20	0.19	0.17	N.D.	1.34
	Styrene	0%	<0.01	0.01	N.D.	N.D.	0.12
	*o*-Xylene	87%	0.06	0.06	0.05	N.D.	0.36

N.D.: not detected.

**Table 6:** Statistical summaries of carbonyl compounds at different sites of the present study.

Sites	Carbonyl compounds	Detection frequency	Mean (ppb)	S.D. (ppb)	Median (ppb)	Min (ppb)	Max (ppb)
YI (*n* = 80)	Formaldehyde	96%	7.40	5.74	4.81	N.D.	26.93
	Acetaldehyde	96%	2.75	2.02	2.24	N.D.	9.89
	Propionaldehyde	60%	0.29	0.40	0.18	N.D.	2.67
	Methyl ethyl ketone	74%	2.57	3.73	1.06	N.D.	15.92
	Butyraldehyde	5%	0.06	0.34	N.D.	N.D.	2.67
	*i*-Valeraldehyde	11%	0.03	0.09	N.D.	N.D.	0.50
	*n*-Valeraldehyde	31%	0.11	0.22	N.D.	N.D.	0.95

YR (*n* = 78)	Formaldehyde	99%	8.06	6.27	5.88	N.D.	31.38
	Acetaldehyde	97%	3.43	3.05	2.10	N.D.	15.74
	Propionaldehyde	50%	0.35	0.61	0.06	N.D.	2.97
	Methyl ethyl ketone	73%	2.05	2.93	0.96	N.D.	13.73
	Butyraldehyde	29%	0.56	1.25	N.D.	N.D.	6.08
	*i*-Valeraldehyde	12%	0.03	0.10	N.D.	N.D.	0.46
	*n*-Valeraldehyde	26%	0.05	0.11	N.D.	N.D.	0.48

GI (*n* = 79)	Formaldehyde	99%	5.99	4.55	4.89	N.D.	30.52
	Acetaldehyde	99%	1.97	1.12	1.85	N.D.	5.35
	Propionaldehyde	44%	0.14	0.18	N.D.	N.D.	0.64
	Methyl ethyl ketone	91%	2.74	3.12	2.04	N.D.	20.56
	Butyraldehyde	4%	0.02	0.08	N.D.	N.D.	0.51
	*i*-Valeraldehyde	8%	0.02	0.06	N.D.	N.D.	0.39
	*n*-Valeraldehyde	28%	0.07	0.13	N.D.	N.D.	0.54

GR (*n* = 80)	Formaldehyde	100%	7.15	5.54	5.45	1.65	28.66
	Acetaldehyde	100%	2.23	1.68	1.86	0.13	12.44
	Propionaldehyde	34%	0.17	0.47	N.D.	N.D.	3.92
	Methyl ethyl ketone	86%	2.29	3.02	1.86	N.D.	22.34
	Butyraldehyde	1%	0.01	0.05	N.D.	N.D.	0.44
	*i*-Valeraldehyde	4%	0.01	0.06	N.D.	N.D.	0.40
	*n*-Valeraldehyde	11%	0.03	0.08	N.D.	N.D.	0.37

SC (*n* = 80)	Formaldehyde	98%	6.52	4.84	5.30	N.D.	25.81
	Acetaldehyde	98%	2.54	2.87	1.48	N.D.	18.38
	Propionaldehyde	38%	0.21	0.43	N.D.	N.D.	2.91
	Methyl ethyl ketone	78%	2.05	2.11	1.75	N.D.	8.59
	Butyraldehyde	10%	0.10	0.43	N.D.	N.D.	2.77
	*i*-Valeraldehyde	5%	0.03	0.15	N.D.	N.D.	0.84
	*n*-Valeraldehyde	18%	0.05	0.12	N.D.	N.D.	0.58

N.D.: not detected.

**(a):** 

Sites	Compounds	Benzene	Ethyl acetate	MIBK	Toluene	*m*, *p*-Xylenes	Styrene
YI	Ethyl acetate	0.053					
	MIBK	0.317∗	0.059				
	Toluene	0.545∗	0.070	0.581∗			
	*m*, *p*-Xylenes	0.532∗	0.155∗	0.753∗	0.692∗		
	Styrene	0.278∗	0.051	0.267∗	0.408∗	0.396∗	
	*o*-Xylene	0.410∗	0.084	0.773∗	0.644∗	0.857∗	0.430∗

YR	Ethyl acetate	0.262∗					
	MIBK	0.114	0.402∗				
	Toluene	0.278∗	0.178∗	0.284∗			
	*m*, *p*-Xylenes	0.380∗	0.601∗	0.475∗	0.212∗		
	Styrene	0.367∗	0.256∗	0.329∗	0.425∗	0.302∗	
	*o*-Xylene	0.385∗	0.644∗	0.445∗	0.269∗	0.949∗	0.349∗

GI	Ethyl acetate	0.027					
	MIBK	−0.016	0.220∗				
	Toluene	0.413∗	0.187∗	0.391∗			
	*m*, *p*-Xylenes	0.032	−0.031	0.300∗	0.638∗		
	Styrene	−0.006	−0.011	0.327∗	0.450∗	0.454∗	
	*o*-Xylene	0.027	−0.028	0.293∗	0.646∗	0.986∗	0.458∗

GR	Ethyl acetate	0.201∗					
	MIBK	0.143∗	0.285∗				
	Toluene	0.416∗	0.490∗	0.343∗			
	*m*, *p*-Xylenes	0.466∗	0.449∗	0.329∗	0.677∗		
	Styrene	0.159∗	0.011	−0.019	0.219∗	0.224∗	
	*o*-Xylene	0.473∗	0.466∗	0.288∗	0.651∗	0.953∗	0.192∗

SC	Ethyl acetate	0.271∗					
	MIBK	0.159∗	0.193∗				
	Toluene	0.645∗	0.217∗	0.230∗			
	*m*, *p*-Xylenes	0.500∗	0.256∗	0.131∗	0.735∗		
	Styrene	0.158∗	0.024	0.706∗	0.241∗	0.117	
	*o*-Xylene	0.511∗	0.310∗	0.190∗	0.718∗	0.978∗	0.151∗

*Correlation coefficients are significant at a level of 0.05.

**(b):** 

Sites	Compounds	SO_2_	PM_10_	O_3_	NO_2_	CO	Temp. (°C)	Wind speed (m/s)
YI	Benzene	−0.074	0.130∗	−0.294∗∗	0.253∗	0.156∗	−0.144∗	−0.214∗
	Ethyl acetate	−0.048	0.144∗	0.052	0.081	−0.057	0.079	−0.109
	MIBK	0.101	0.162∗	−0.027	0.367∗	−0.064	0.063	−0.266∗
	Toluene	0.053	0.254∗	−0.077	0.463∗	0.024	0.048	−0.297∗
	*m*, *p*-Xylenes	0.030	0.235∗	−0.121	0.377∗	−0.038	0.063	−0.297∗
	Styrene	0.196∗	0.188∗	−0.239∗∗	0.199∗	−0.022	0.223∗	−0.253∗
	*o*-Xylene	0.048	0.140∗	−0.127	0.302∗	−0.079	0.191∗	−0.330∗

YR	Benzene	0.187∗	0.317∗	−0.424∗	0.461∗	0.592∗	−0.370∗	0.053
	Ethyl acetate	0.412∗	0.341∗	−0.370∗	0.462∗	0.290∗	−0.060	−0.289∗
	MIBK	0.333∗	0.133	−0.207∗	0.280∗	0.081	0.022	−0.063
	Toluene	0.013	0.141	−0.220∗	0.217∗	0.166∗	−0.087	0.098
	*m*, *p*-Xylenes	0.589∗	0.322∗	−0.473∗	0.552∗	0.428∗	0.003	−0.373∗
	Styrene	−0.013	0.093	−0.287∗	0.316∗	0.351∗	−0.112	−0.069
	*o*-Xylene	0.516∗	0.323∗	−0.468∗	0.542∗	0.373∗	0.011	−0.362∗

GI	Benzene	0.586∗	0.367∗	0.098	0.385∗	0.070	0.213∗	−0.069
	Ethyl acetate	0.186∗	0.151∗	0.144∗	0.195∗	0.155∗	0.020	−0.031
	MIBK	0.106	0.176∗	−0.156∗	0.228∗	0.357∗	−0.122	−0.031
	Toluene	0.316∗	0.302∗	−0.047	0.375∗	0.122	0.154∗	−0.035
	*m*, *p*-Xylenes	0.019	0.057	−0.076	0.061	0.105	−0.057	0.027
	Styrene	0.063	0.123	−0.078	0.154∗	0.164∗	−0.047	0.011
	*o*-Xylene	0.021	0.060	−0.082	0.074	0.120	−0.074	0.042

GR	Benzene	0.504∗	0.423∗	−0.112	0.558∗	0.448∗	−0.017	−0.185∗
	Ethyl acetate	0.311∗	0.334∗	−0.105	0.384∗	0.249∗	−0.048	−0.206∗
	MIBK	0.265∗	0.173∗	−0.141∗	0.275∗	0.241∗	−0.056	−0.060
	Toluene	0.521∗	0.360∗	−0.137	0.566∗	0.480∗	0.016	−0.315∗
	*m*, *p*-Xylenes	0.430∗	0.362∗	−0.483∗	0.672∗	0.591∗	−0.199∗	−0.355∗
	Styrene	0.107	0.053	−0.053	0.150∗	0.131	−0.056	−0.090
	*o*-Xylene	0.384∗	0.402∗	−0.517∗	0.699∗	0.584∗	−0.192∗	−0.369∗

SC	Benzene	0.545∗	0.512∗	−0.086	0.430∗	0.426∗	−0.108	−0.080
	Ethyl acetate	0.218∗	0.249∗	0.013	0.172∗	0.175∗	−0.069	−0.007
	MIBK	0.177∗	0.063	−0.034	0.068	0.106	−0.075	−0.011
	Toluene	0.267∗	0.173∗	−0.188∗	0.274∗	0.218∗	−0.006	−0.158∗
	*m*, *p*-Xylenes	0.117	0.129∗	−0.262∗	0.325∗	0.126	0.190∗	−0.367∗
	Styrene	0.082	0.061	−0.020	0.046	0.118	−0.056	−0.010
	*o*-Xylene	0.127∗	0.163∗	−0.241∗	0.332∗	0.119	0.228∗	−0.361∗

*Correlation coefficients are significant at a level of 0.05.

**(a):** 

Sites	Compounds	FRML	ACTL	PPNL	MEK	BTL	iVAL
YI	ACTL	0.838∗					
	PPNL	0.356∗	0.455∗				
	MEK	0.507∗	0.320∗	−0.151			
	BTL	0.144	0.274∗	0.164	−0.061		
	iVAL	0.099	0.074	0.155	0.077	−0.053	
	VAL	0.074	0.148	0.149	0.168	−0.063	−0.039

YR	ACTL	0.821∗					
	PPNL	0.638∗	0.796∗				
	MEK	0.363∗	0.209	0.061			
	BTL	0.287∗	0.205	0.096	−0.014		
	iVAL	0.328∗	0.287∗	0.385∗	0.385∗	−0.069	
	VAL	0.397∗	0.301∗	0.032	−0.009	0.340∗	0.189

GI	ACTL	0.428∗					
	PPNL	0.266∗	0.485∗				
	MEK	0.064	0.022	−0.063			
	BTL	0.439∗	0.293∗	0.041	−0.081		
	iVAL	0.011	0.050	0.001	0.356∗	0.120	
	VAL	0.271∗	0.141	0.303∗	0.389∗	−0.102	0.245∗

GR	ACTL	0.584∗					
	PPNL	0.452∗	0.697∗				
	MEK	0.088	0.001	−0.057			
	BTL	−0.028	0.004	−0.040	0.020		
	iVAL	0.040	0.045	−0.046	0.029	−0.021	
	VAL	0.123	0.095	0.059	0.057	−0.039	0.179

SC	ACTL	0.271∗					
	PPNL	0.120	0.713∗				
	MEK	0.425∗	0.251∗	−0.123			
	BTL	−0.066	−0.113	−0.091	−0.171		
	iVAL	−0.054	0.426∗	0.166	0.091	−0.042	
	VAL	0.122	−0.118	0.004	−0.229∗	0.044	−0.088

*Correlation coefficients are significant at a level of 0.05.

FRML: formaldehyde; ACTL: acetaldehyde; PPNL: propionaldehyde; MEK: methyl ethyl ketone; BTL: butyraldehyde.

iVAL: *i*-valeraldehyde; VAL: *n*-valeraldehyde.

**(b):** 

Sites	Compounds	SO_2_	PM_10_	O_3_	NO_2_	CO	Temp (°C)	Wind speed (m/s)
YI	FRML	0.225∗	0.298∗	0.532∗	0.016	−0.175	0.276∗	−0.160
	ACTL	0.448∗	0.365∗	0.279∗	0.230∗	0.093	0.021	−0.072
	PPNL	0.345∗	0.076	0.053	0.168	0.126	0.051	−0.074
	MEK	−0.089	0.139	0.237∗	−0.023	−0.176	0.134	−0.267∗
	BTL	0.575∗	0.167	0.130	−0.019	−0.007	0.014	0.236∗
	iVAL	−0.016	0.014	0.275∗	−0.125	−0.019	0.090	0.046
	VAL	−0.012	0.099	−0.049	0.072	0.110	−0.239∗	0.049

YR	FRML	0.096	0.509∗	0.115	0.498∗	0.185	0.151	−0.246∗
	ACTL	0.143	0.581∗	−0.112	0.714∗	0.402∗	−0.102	−0.247∗
	PPNL	0.084	0.337∗	−0.099	0.497∗	0.275∗	−0.079	−0.251∗
	MEK	0.162	0.169	−0.221	0.086	0.100	0.004	−0.097
	BTL	−0.105	0.227∗	0.144	0.100	−0.038	0.101	0.220
	iVAL	0.033	0.062	−0.157	0.090	0.084	0.055	−0.173
	VAL	0.035	0.297∗	0.107	0.209	0.049	0.251∗	−0.172

GI	FRML	0.033	0.118	0.279∗	0.126	−0.049	0.362∗	−0.127
	ACTL	0.261∗	0.410∗	0.058	0.525∗	0.332∗	0.249∗	−0.303∗
	PPNL	0.139	0.091	0.194	0.169	0.026	0.528∗∗	0.000
	MEK	−0.247∗	−0.062	−0.195	−0.195	−0.073	0.132	−0.030
	BTL	0.039	0.051	0.155	0.176	−0.049	0.116	−0.041
	iVAL	−0.063	0.119	−0.028	0.038	−0.004	0.066	−0.087
	VAL	−0.163	0.039	−0.015	−0.128	−0.150	0.312∗	0.021

GR	FRML	−0.004	0.161	0.180	0.100	0.027	0.248∗	−0.197
	ACTL	0.258∗	0.505∗	0.044	0.478∗	0.360∗	0.114	−0.392∗
	PPNL	0.004	0.072	0.141	0.069	0.007	0.200	−0.174
	MEK	0.042	0.067	−0.218	0.072	0.084	0.056	−0.079
	BTL	−0.081	−0.021	−0.048	−0.096	−0.072	−0.024	−0.064
	iVAL	0.052	0.205	0.089	0.095	−0.004	0.129	−0.072
	VAL	0.075	0.035	0.110	−0.037	0.057	0.329∗	−0.072

SC	FRML	0.347∗	0.414∗	0.015	0.460∗	0.377∗	0.138	−0.378∗
	ACTL	0.295∗	0.327∗	−0.109	0.466∗	0.348∗	−0.057	−0.405∗
	PPNL	0.292∗	0.223∗	−0.133	0.263∗	0.155	−0.127	−0.244∗
	MEK	0.267∗	0.465∗	−0.063	0.488∗	0.590∗	−0.231∗	−0.277∗
	BTL	−0.135	−0.061	0.075	−0.138	−0.155	0.116	0.097
	iVAL	0.058	0.025	−0.283∗	0.252∗	0.300∗	−0.175	−0.237∗
	VAL	−0.162	−0.120	0.189	−0.196	−0.198	0.396∗	0.026

*Correlation coefficients are significant at a level of 0.05.

FRML: formaldehyde; ACTL: acetaldehyde; PPNL: propionaldehyde; MEK: methyl ethyl ketone; BTL: butyraldehyde.

iVAL: *i*-valeraldehyde; VAL: *n*-valeraldehyde.

**Table 9:** Comparison of the concentrations (ppb) of VOC reported in present method with other cities in the world.

S. No.	City/country	Sampling period	Site characteristics	*N*	Analysis	BZ	TOL	XYLN	MIBK	EA	STR	T/B ratio	Reference
1	Pohang/Korea∗	Jan, 2006–Feb, 2009	Steel, Ferro-alloy industries	—	On-line TD/GC-FID	0.12	0.18	0.13	—	—	0.34	1.50	[18]
			Residential			0.78	2.50	0.66	—	—	0.14	3.21	

2	Yokohoma/Japan	June 2007–Nov, 2008	Petrochemical industries	69	GC-FID	0.70~2.10	1.60~5.20	0.40~2.10	—	0.40~0.60	—	—	[19]
			Residential			0.40~0.90	1.90~2.20	0.20~0.70	—	0.60	—	—	

3	Tzouying/Taiwan	Oct-Nov, 2004	Oil refinery	24	GC-MS	1.6~2.5	24.5~42.00	1.5~2.10	—	—	—	—	[20]

4	Kaohsiung/Taiwan	July and Oct, 2003	Petrochemical industrial park (Rush hours)	—	GC-MS	11.29	160.88	22.07	—	—	15.16	14.25	[21]
			Nonrush hours			13.49	101.99	15.15	—	—	—	7.56	

5	Southern Taiwan, Ping-Nan IP	July, 2003–Dec, 2004	Iron and steel industries	129	GC-FID	1.98	4.33	3.72	—	—	—	2.18	[22]
	Rem-Wu IP		Petrochemical and general manufacturing industries			1.99	14.52	7.14	—	—	—	7.29	
	Naan-Zi IP		Petroleum and petrochemical industries			2.17	5.41	4.95	—	—	—	2.49	

6	Shanghai/China	Jan, 2007–Mar, 2010	Chemical and steel industries	284	GC-MS	1.81	4.70	1.89	—	2.09	0.14	2.59	[23]

7	Mumbai/India∗	2004	Petrochemical, pharma, and plastic industries	—	TD/GC-MS	0.62	1.47	0.02	—	—	0.03	2.37	[24]

8	Mumbai/India∗	May 2001–Apr, 2002	Petrochemical industries	24	TD/GC-MS	60.98	20.42	0.03	—	—	—	0.33	[25]
			Residential			13.71	7.48	0.25	—	—	—	0.55	

9	Dunkerque/France∗	July–Sep, 2009	Petrochemical, chemical, and steel industries	—	GC-FID	0.12	0.31	0.19	—	—	0.03	2.58	[26]

10	Dunkerque/France∗	Jan, 2007	Petrochemical, chemical, and steel industries	—	TD/GC-FID	0.58	2.59	0.63	—	—	—	4.46	[27]
		June, 2007				0.47	0.54	0.18	—	—	—	1.14	

11	Seoul/Korea	Feb–Jul, 2009	Urban residential	18	GC-MS	0.58	5.39	1.16	—	—	0.45	9.29	[28]

12	Yeosu industrial/Korea	May, Aug, Oct 2008, Jan 2009	Oil refinery and [petrochemical industries	234	TD/GC-MS	3.65	3.25	3.08	0.11	0.39	0.25	0.89	this study
	Yeosu residential/Korea		Residential	160	TD/GC-MS	1.07	1.61	0.46	0.01	0.11	0.15	1.50	
	Gwangyang industrial/Korea		Steel industries	239	TD/GC-MS	1.64	0.97	1.37	0.01	0.12	0.01	0.59	
	Gwangyang residential/Korea		Residential	199	TD/GC-MS	0.59	0.98	0.48	<0.01	0.10	<0.01	1.66	
	Suncheon comparison/Korea		Residential	240	TD/GC-MS	0.38	0.68	0.26	<0.01	0.07	<0.01	1.79	

*N*: no. of data; TD: thermal desorption; BZ: benzene; TOL: toluene; XYLN: *m*, *p*, *o*-xylenes; STR: styrene; EA: ethyl acetate; STR: styrene; IP: industrial park.∗Concentration of VOC is reported by those authors in *μ*g/m^3^ and in present table they are converted into ppb (at 15°C).

**Table 10:** Comparison of the concentrations of carbonyl compounds reported in present method with other cities in the world.

S. No.	City/country	Sampling period	Site characteristics	*N*	FRML	ACTL	PPNL	BTL	iVAL	nVAL	MEK	*C*_1_/*C*_2_	Reference
1	Kolkata/India	Site 1, summer	Mixed (res., traffic, and ind.)	—	18.60	9.09	1.50	1.46	—	—	—	2.0	[29]
		Winter			13.76	6.76	1.02	0.75	—	—	—	2.0	
		Site 2, summer	Mixed (res., traffic, and ind.)		11.02	4.59	0.73	1.30	—	—	—	2.4	
		Winter			9.92	4.98	0.95	0.74	—	—	—	2.0	

2	Guangzhou/China	July–Sep, 2003	Residential	5	10.88	5.83	1.11	0.66	0.23	0.25		1.9	[30]
			Industrial	5	11.07	3.96	0.55	0.29	0.08	0.62		2.8	

3	Guangzhou/China	17–19, Nov–2005 (clear days)	Residential and traffic site	12	4.74	3.95	0.35	0.78	0.12	0.16		1.2	[31]
		30 Nov–2, Dec 2005 (hazy days)	Residential and traffic	12	11.49	8.91	1.16	0.81	0.18	2.74		1.3	

4	Ansan, Shi Hung/Republic of Korea	Aug, 2004–Sep, 2005	Industrial	—	19.30	19.50	19.00	13.00	0.93	0.82		1.0	[8]

5	Gumi/Republic of Korea	Dec, 2003–Nov, 2004	Industrial site 1	172	3.40	7.35	0.80				2.40	0.5	[6]
		2004	Industrial site 2	78	3.87	2.52	1.01				4.81	1.5	
		2004	Industrial, site 3	77	3.86	4.22	1.04				3.32	0.9	

6	Mexico city/Mexico	Mar,1998–Feb, 1999	Mixed (res., com., and ind.)	35	4.33	2.31	0.53	0.29	0.44	0.16		1.9	[32]

7	Rome/Italy	2006	Urban	—	2.16	1.26	0.41	1.61	0.06	0.13		1.7	[33]

8	Kurkimaki/Finland	—	Residential	—	0.37	0.36	0.05	0.03				1.0	[34]

9	Roverdo	Nov-Dec, 2005	Rural	—	—	0.38	0.10	0.05			0.08	—	[35]
	Zurich/Switzerland	Nov-Dec, 2005	Urban res.	—	—	0.86	0.12	0.04			0.03	—	

10	Santiago/Chile	Nov-Dec, 2003	Downtown	40	3.90	3.00	0.46	3.30	0.11			1.3	[36]

11	Yeosu industrial/Korea	May, Aug, Oct 2008, Jan 2009	Oil refinery, petrochemical industries	80	7.40	2.75	0.29	0.06	0.03	0.11	2.57	2.7	This study
	Yeosu residential/Korea		Residential	78	8.06	3.43	0.35	0.56	0.03	0.05	2.05	2.3	
	Gwangyang industrial/Korea		Steel industries	79	5.99	1.97	0.14	0.02	0.02	0.07	2.74	3.0	
	Gwangyang residential/Korea		Residential	80	7.15	2.23	0.17	0.01	0.01	0.03	2.29	3.2	
	Suncheon comparison/Korea		Residential	80	6.52	2.54	0.21	0.10	0.03	0.05	2.05	2.6	
